# Family physicians’ perspectives on the impact of COVID-19 on preventative care in primary care: findings from a qualitative study

**DOI:** 10.1093/fampra/cmac113

**Published:** 2022-10-21

**Authors:** Crystal Vaughan, Julia Lukewich, Maria Mathews, Emily Gard Marshall, Lindsay Hedden, Sarah Spencer, Dana Ryan, Rita K McCracken, Paul Gill, Stephen Wetmore, Richard Buote, Leslie Meredith, Lauren Moritz, Judith Belle Brown

**Affiliations:** Faculty of Nursing, Memorial University of Newfoundland, St John’s, Canada; Faculty of Nursing, Memorial University of Newfoundland, St John’s, Canada; Department of Family Medicine, Schulich School of Medicine and Dentistry, Western University, London, Canada; Department of Family Medicine, Dalhousie University, Halifax, Canada; Faculty of Health Sciences, Simon Fraser University, Burnaby, Canada; Faculty of Health Sciences, Simon Fraser University, Burnaby, Canada; Faculty of Nursing, Memorial University of Newfoundland, St John’s, Canada; Department of Family Medicine, Providence Health Care, Vancouver, Canada; Department of Family Practice, University of British Columbia Faculty of Medicine, Vancouver, Canada; Temerty Faculty of Medicine, Department of Family & Community Medicine, University of Toronto, Toronto, Canada; Gateway Centre of Excellence in Rural Health, Gateway Rural Health Institute, Goderich, Canada; Department of Family Medicine, Schulich School of Medicine and Dentistry, Western University, London, Canada; Department of Family Medicine, Dalhousie University, Halifax, Canada; Department of Family Medicine, Schulich School of Medicine and Dentistry, Western University, London, Canada; Department of Family Medicine, Dalhousie University, Halifax, Canada; Department of Family Medicine, Schulich School of Medicine and Dentistry, Western University, London, Canada

**Keywords:** COVID-19, family practice, pandemic, preventative care, primary care physician

## Abstract

**Introduction:**

Health system disruptions, caused by unexpected emergencies such as disease outbreaks, natural disasters, and cybercrimes, impact the delivery of routine preventative care. As comprehensive care providers, family physicians (FPs) devote significant time to prevention. However, without emergency and pandemic plans in place in primary care, FPs face added barriers to prioritizing and sustaining preventative care when health systems are strained, which was evident during the COVID-19 pandemic. This study aims to describe FPs’ experiences providing preventative care during the COVID-19 pandemic and their perceptions of the impacts of disrupted preventative care in primary care settings.

**Methods:**

Using a qualitative descriptive approach, we conducted semistructured interviews with FPs across 4 provinces in Canada (i.e. Newfoundland and Labrador, Nova Scotia, Ontario, British Columbia) between October 2020 and June 2021 as part of a larger multiple case study. These interviews broadly explored the roles and responsibilities of FPs during the COVID-19 pandemic. Interviews were coded thematically and codes from the larger study were analysed further using an iterative, phased process of thematic analysis.

**Results:**

Interviews averaged 58 min in length (range 17–97 min) and FPs had a mean of 16.9 years of experience. We identified 4 major themes from interviews with FPs (*n* = 68): (i) lack of capacity and coordination across health systems, (ii) patient fear, (iii) impacts on patient care, and (iv) negative impacts on FPs. Physicians voiced concerns with managing patients’ prevention needs when testing availability and coordination of services was limited. Early in the pandemic, patients were also missing or postponing their own primary care appointments. Change in the provision and coordination of routine preventative care had negative impacts on both patients and physicians, affecting disease incidence/progression, physician workload, and psychological wellbeing.

**Conclusion:**

During the COVID-19 pandemic, upstream care efforts were impacted, and FPs were forced to reduce their provision of preventative care. FPs contribute direct insight to primary care delivery that can support pandemic planning to ensure preventative care is sustained during future emergencies.

**Key Messages**

- COVID-19 caused FPs to reduce their provision of preventative care.
- System issues limited preventative care, impacting both patients and FPs.
- FP perspectives are needed to inform pandemic plans.
- Pandemic plans could help sustain preventative care when healthcare is disrupted.

## Background

Preventative care is a key component of routine healthcare delivery in primary care. Health system disruptions resulting from natural disasters,^1^ cybercrimes, and disease outbreaks disrupt the delivery of routine prevention services in primary care. Since 2020, Canada has seen these disruptions emerge during region-wide flooding in British Columbia (BC), wildfires across BC and Alberta, the cyberattack of Newfoundland and Labrador’s (NLs) healthcare system,^2–5^ as well as the COVID-19 pandemic. Consistently, there were unexpected shifts in resource utilization and availability which directly impacted the delivery of routine primary care services. In particular, the COVID-19 pandemic placed significant strain on health systems at large and caused disruptions that had noticeable impacts on preventative care.^6,7^ This global crisis emphasized the need for preparation and planning to ensure the provision of preventative care continues and is prioritized in the presence of future emergencies and secondary health system disruptions.

While preexisting and emerging pandemic preparedness documents emphasize the need to continue the delivery of primary healthcare services,^8–10^ a lack of preparation and planning in primary care prior to and during the COVID-19 pandemic led to a reduction in preventative care. Community-level prevention efforts, to support upstream public healthcare, were absent which had a direct impact on family physicians’ (FPs’) ability to deliver preventative care at the individual level. By January 2021, nearly 1 year into the pandemic, cancer screening rates in Canada remained 20%–35% lower than prepandemic rates.^11^ Notably, the provision and coordination of preventative care rely upon FPs. FPs are expected to spend 20% of their total annual time,^12^ or 4.4 h per working day (for adult patients),^13^ on prevention, despite competing demands in practice and global workforce shortages which inevitably impacts their ability to deliver preventative care under ordinary circumstances.^14^ When natural disasters, health crises, or other emergencies create additional challenges and resources are stretched, FPs are under even further strain which can influence their ability to prioritize and deliver preventative care optimally, if at all.

It is critical to examine how structural and organizational limitations during the COVID-19 pandemic impacted the provision and coordination of preventative care. In particular, FPs should inform the development of plans to ensure preventative care is sustained in future emergencies and unplanned health system disruptions. The purpose of this study is to describe FPs’ experiences providing preventative care during the COVID-19 pandemic and their perceptions of the impacts of disrupted preventative care in primary care.

## Methods

### Design

This study was part of a larger study that employed multiple mixed-methods case studies using policy analyses and qualitative interviews across 4 Canadian provinces (i.e. NL, Nova Scotia [NS], Ontario [ON], BC). The overall aims of this larger study were to understand the proposed, actual, and potential roles of FPs during the COVID-19 pandemic; the facilitators and barriers that impacted FP roles during this time; and the influence gender had on FPs’ ability to enact certain roles. All aims were analysed across selected provinces. This study aligns with pragmatism as a research paradigm that values clinically relevant interventions/findings, discovered through the use of qualitative and/or quantitative methodologies.^15^ A detailed description of the study protocol has been published elsewhere.^16^ For the purpose of this study and the data presented in this paper, a qualitative descriptive approach was used (as only the qualitative interviews were analysed).

### Setting and participants

We included 4 regions (i.e. Eastern Health region of NL, the province of NS, ON Health West, Vancouver Coastal health region in BC) from 4 provinces. We selected these regions for the diversity they provide in COVID-19 numbers and representation of urban/rural communities, among other factors.^16^ Healthcare in Canada is primarily regulated by provinces/territories with jurisdictional differences across regions within provinces. Primary care is delivered through public or private practice and covered by provincial, single-payer public health insurance plans.^17^ In Canada, FPs can opt for a variety of remuneration schemes such as fee-for-service and alternate payment plans (e.g. global funding, capitation, salary)^18^; however, availability varies across provinces.

We recruited FPs using multiple strategies (e.g. institutional/organizational member lists, social media, snowball sampling) aiming to achieve maximum variation in FP characteristics (e.g. practice type, community size).^19^ FPs were included if they were licensed by their provincial/territorial medical regulatory authority in 2020 and were practicing or eligible to be clinically active in the region of study. Sampling continued until FP perspectives were well represented and data saturation was obtained.

### Data collection and analysis

We conducted semistructured, qualitative interviews from October 2020 to June 2021 with eligible FPs across 4 regions. Designated research team members (SS, DR, RB, LMe, and LMo) completed interviews virtually (i.e. video conference or telephone) using a standardized interview guide. Verbal consent was obtained from each FP at the time of their interview. Interviews were anonymized, transcribed verbatim, and read independently by at least 2 members of our research team to make cross-case comparisons. We developed a robust coding framework using transcripts and additional field notes collected by the interviewers; transcripts were then analysed using NVivo V.12 (QSR International). We performed thematic analysis^20,21^ across transcripts and node reports from the initial analysis to identify recurring topics and associated themes. This analytic approach included 6 phases whereby transcripts and/or node reports were repeatedly reviewed to develop initial ideas; and codes were generated and collapsed into possible themes. Themes were reviewed to ensure they represented data in the interview transcripts. Finally, themes were titled/defined to allow for reporting/dissemination of analysis.

### Reflexivity

FPs included on this project were knowledge users and collaborative partners (RKM, PG, and SW). Team members responsible for data collection and analysis (CV, JL, MM, EGM, LH, SS, DR, RB, LMe, and LMo) are not FPs, rather trained health services researchers, reducing the potential for confirmation bias. By ensuring reflexivity among team members, we were able to identify actionable messages from FP perspectives and capture the true organization and context of primary care during the COVID-19 pandemic.

## Results

Interviews were completed by 68 FPs with the following provincial breakdown: NS = 21, ON = 20, BC= 15, NL = 12. This was a heterogeneous sample with different demographic profiles, practicing across both urban and rural areas (Table 1).^22^ Interviews ranged from 17 to 97 min and averaged 58 min in length.

**Table 1.** Characteristics of 68 family physician participants by province (2020–2021).

	Ontario *n* = 20 *n* (%)	Nova Scotia *n* = 21 *n* (%)	British Columbia *n* = 15 *n* (%)	Newfoundland and Labrador *n* = 12 *n* (%)	Total *n* = 68 *n* (%)
Gender^a^					
Men	10 (50)	9 (42.9)	4 (36.4)	4 (33.3)	27 (39.7)
Women	10 (50)	12 (57.1)	11 (63.6)	8 (66.7)	41 (60.3)
Practice type					
Fee-for-service	4 (20)	7 (33.3)	6 (40)	5 (41.7)	22 (32.4)
Alternative payment plan^b^	16 (80)	14 (66.7)	9 (60)	7 (58.3)	46 (67.6)
Hospital privileges					
No	15 (75)	6 (28.6)	3 (20)	5 (41.7)	18 (26.5)
Yes	5 (25)	15 (71.4)	12 (80)	7 (58.3)	49 (73.5)
Community size^c^					
Rural	9 (45)	8 (38.1)	0	3 (25)	20 (29.4)
Small urban	1 (5)	0 (0)	0	0 (0)	1 (1.5)
Urban	8 (40)	13 (61.9)	15 (100)	8 (66.7)	44 (64.7)
Mix	2 (10)	0 (0)	0	1 (8.3)	3 (4.4)
Years in practice (mean)	18.7	15.4	16.9	16.3	16.9

^a^Gender was asked as an open-ended question.

^b^Alternate payment includes all non-fee-for-service or enhanced fee-for-service payment types.

^c^Rural <10,000 population, small urban = 10,000–99,999 population, urban >1,000,000.

We identified 4 major themes related to preventative care from interview data: (i) lack of capacity and coordination across health systems, (ii) patient fear, (iii) impacts on patient care, and (iv) negative impacts on FPs.

### Lack of capacity and coordination across health systems

FPs interviewed felt that a lack of system capacity and coordination for preventative care services during the pandemic limited their ability to provide routine preventative care. Laboratories and diagnostic imaging departments reduced their routine/non-urgent services so that many preventative tests (e.g. Pap smears) could not be ordered/completed:

[…] there were some sort of preventative health things that we couldn’t offer because we couldn’t send a Pap swab in. And for a while too we were encouraged not to order routine bloodwork … so if a diabetic was due for a cholesterol check and an A1C, we weren’t allowed to order it. [NS02]

FPs noted that certain routine screening programmes (e.g. faecal immunochemical test) had suspended operations and were not providing at-home screening kits to patients: “*… with the FOBT [fecal occult blood testing] testing so that’s to look at blood in the stool. That type of testing … they were no longer processing, so they stopped that, so that prevention went out*” [ON08].

When these programmes and services resumed normal operations, they deferred responsibility to patients and FPs to ensure that screening was up to date, with limited efforts to manage the backlog generated from extended closures: “*… the colon cancer screening program not only didn’t function for a number of months, but when they reopened, they opted not to contact the patients that missed their screening window during those six months … they just are never going to*” [NS15]. Moreover, there was little coordination and communication with FPs and patients about how missed screening would be addressed: “*They didn’t tell people. They didn’t tell you. I read it in an email … You should call them up and get them to mail [the test] to you*” [NS15]. Therefore, early in the pandemic, certain preventative screening tests were often missed unless patients or FPs were proactive in their management and coordination of preventative care.

Laboratories and diagnostic imaging facilities, once reopened, provided limited communication to FPs about how to access their services:

[…] we didn’t know … what services were being opened up when like, blood collection and how diagnostics were going down … we would get a fax saying, this is the way … to order diagnostics now, or … order bloodwork … it would be like a general letter faxed to the office, there was no direct communication in terms of planning or those type things. [NL11]

In some cases, FPs created screening-specific clinics to ensure the backlog of missed or delayed tests was addressed and routine tests were completed once in-person care resumed: “*… when we were sort of given the okay to restart Paps … we started running Pap clinics in the evenings*” [ON12].

### Patient fear

During the pandemic, FPs noted that patients’ feelings of fear and related behavioural changes impacted their receipt of preventative care. Patients avoided or missed routine appointments because of their fear of contracting COVID-19 from healthcare workers or patients in waiting rooms: “*… a lot of patients didn’t want to be seen in person because they didn’t want to catch it [COVID-19 infection] themselves in the cesspool they imagined their doctor’s office to be*” [NL08]. FPs explained how patients’ fear could lead them to: “… *avoiding care or coming in because they’re worried about it exposing them to people in the waiting room that might have COVID*” [NS01].

FPs observed how patients mistakenly believed that primary care practices were closed: “*And some patients did say, ‘Oh, well, I didn’t do this because I thought you were closed’ … I didn’t follow up with you*” [NS17]. FPs made concerted, proactive efforts to inform and reassure their patients that primary care services were available for either virtual or in-person visits:

[…] people had a sense, especially at the beginning of the pandemic that the clinic was closed … which was not the case … so we learned that we need to inform our patients that the clinic is still open … if you really feel like there’s something personal that you need to talk to somebody face-to-face … please let us know, we can make that happen. [NS05]

In addition, FPs had to reassure patients that an in-person visit was safe, while reminding patients of the benefits of preventative care appointments and the impact it can have on their future healthcare status: “*We actually had to advocate and encourage people to come still for their prenatal appointments, and their well-baby appointments and things like that. Like they know that we’re here, we’re safe, we got this figured out, please still come*” [NS02].

### Impacts on patient care

The suspension of preventative care had negative impacts on patient care. FPs believed that reduced routine preventative care interactions would result in exacerbations of chronic conditions, missed or late diagnoses, and increased high-risk sexual behaviours (and consequences). Without routine monitoring (e.g. blood pressure measurements), FPs noted deterioration in the health status of patients with chronic conditions:

[…] we weren’t checking blood pressures—is a good example, right. Not everybody had a blood pressure monitor at home that they could tell you what their blood pressure is … the local drugstore that had a blood pressure centre where patients would go and measure their pressure, that was taken away with COVID. So, all of a sudden, they’re coming in and now their pressure’s high. Well, they haven’t even done their blood work … so now they have no labs. They haven’t been done for a year. So, what has changed is the complexity of their presenting issues have gone up, and the urgency of dealing with them have gone up. [NS18]

As well, FPs described patients with new cancer diagnoses which were delayed or identified at a later stage due to postponed screening tests when “*the imaging department cancelled the majority of non-urgent diagnostic imaging tests*” [BC05]. A delayed diagnosis due to lack of access to diagnostic services resulted in a terminal illness that could have been prevented:

[…] we have one patient in particular who is now palliative and quite advanced with a liver cancer that presumably would have been picked up at an earlier stage and better able to treat if … her original investigations had been done as planned. [BC05]

Important routine screening, such as mammograms, were often delayed leading some FPs to speculate that the severity of the malignancy may have decreased with early detection:

[…] people who haven’t had mammograms, who have got lesions. Now … we never have the crystal ball to say whether a three-month delay was the difference between them needing treatment A and treatment B. But … there are patients coming forward with malignancy. [NS17]

FPs reported that the reduction in routine visits and opportunities to provide health education and harm reduction supplies for vulnerable populations contributed to an increase in infections and unplanned pregnancies: “*… what we’ve seen in our community is that there’s an increase in HIV regionally… There’s been an increase in hepatitis C as well, there’s been an increase in syphilis, chlamydia, gonorrhoea, there’s been an increase in unplanned pregnancies*” [BC13].

### Negative impacts on FPs

The disruption of preventative care services produced several negative effects for FPs. In addition to increased workloads related to catching up on routine screening (e.g. evening Pap smear clinics described above), FPs felt that reduced preventative care influenced the functionality of their practices and their mental wellbeing.

FPs noted that when patients began presenting for routine appointments again, these visits were longer and more complex because unaddressed, preexisting medical concerns had worsened during the pandemic, mental health concerns were becoming more prevalent, and preventative care screening tests were delayed:

[…] it was super scary; it was like a tsunami, right? It was like an eerie quietness and you’re like, I am going to get hit so hard when people can start showing up again. And sure enough, that fourth wave ... it was like a tsunami. The mental health issues, the untreated medical issues, the ultrasounds and x-rays that didn’t happen, it was really profound. [NL03]

FPs reflected on their distress when they were unable to assess patients in person but had to make decisions about the urgency of patients’ complaints or requests; they attempted to align their decisions with public health recommendations while aiming to maintain a patient-centred approach to care:

The biggest challenge was navigating who comes in the office and when … because you can do blood pressure appointments from home if they have a cuff. But if they don’t have a cuff, they should come in. And then … should they come in if they’ve been perfectly controlled for a really long time … like making those decisions, which are a bit nuanced, and patient specific, were definitely challenges. [NS21]

FPs described assessing the risks and benefits of bringing patients into the office while simultaneously paying attention to workflow issues and patient volumes. FPs felt troubled when attempting to rationalize appointment changes and the idea that in-person visits were not available to all patients, rather those who were considered to have serious concerns which can involve an element of subjectivity:

[…] Pap smear screening, for example … if I am suggesting to people that perhaps it’s not urgent and maybe it’s something that could wait and that we reserve in-person visits for things that are more urgent … that would be difficult for patients to understand when it’s okay to go and get a facial, right? So … we have been struggling at our clinic a lot to decide how we make those decisions […]. [BC01]

Most notably, FPs experienced a great deal of moral distress due to the disruption of preventative care. When FPs felt that they were unable to execute their preferred plan of care based on their own professional assessment, they juggled the options that were available to optimize care:

[…] I still have sick patients, I have well-babies, I have post-partums, I have palliative patients … I know that that care can’t stop … I couldn’t abandon my patients and I also couldn’t tell everybody to go to [the emergency department] with all their concerns because I work at [emergency] and they were doing everything they could to protect that resource. [NL11]

Services that, prior to the pandemic, were available in the community were now inaccessible unless departments (like emergency) were utilized. FPs felt morally distressed about the inappropriate use of emergency resources:

[…] if you need somebody to come back to check an INR [a blood test measuring clotting time] or potassium or creatinine, you can’t wait 4 weeks … I was bringing them back to [the emergency department]. But it’s not ideal … it uses up emergency resources. [NS04]

## Discussion

By interviewing FPs, we were able to describe FPs’ experiences in providing preventative care during the COVID-19 pandemic and their perceptions of the impacts of disrupted preventative care in primary care. The reflections from FPs in this study illustrate how the COVID-19 pandemic significantly impacted the delivery of preventative care in primary care practices. Preventative care as a routine service was not relatively available; therefore, FPs were forced to overcome system-level and patient-related barriers when seeking to provide preventative care. These challenges impacted the incidence and progression of disease among some patients and caused FPs to feel morally distressed and overwhelmed with increased workloads as they navigated the COVID-19 pandemic.

Disruptions to preventative care are reflected in other Canadian studies. In ON, from March to December 2020, primary care visits involving certain preventative services were reduced, with 89% and 16% fewer routine physical exams and well-baby visits, respectively.^23^ As well, a study based on 2020 data in Ottawa, ON reported that screening for cervical cancer, colorectal cancer, and type 2 diabetes decreased by 7.5%, 8.1%, and 4.5%, respectively, during the pandemic, estimating that hundreds of thousands of screening appointments were overdue in ON in 2020.^24^. Similarly, cancer screening rates during the early stages of the pandemic were reduced across the United States^25^ and United Kingdom.^26^

Our study offers important insights into future disaster and pandemic planning. Modulating demand for laboratory and diagnostic services has been identified as a role for FPs during a pandemic^27^ and is an example of the how the gatekeeping role changes during a health system crisis. The successful delivery of preventative care interventions depends upon the alignment of system and organizational factors (among others) with practice-, provider-, and patient-level components, as noted by the P3 Model.^28^ System and organizational factors relate to system- and programme-level attributes. During the COVID-19 pandemic, the availability of laboratory and diagnostic services was disrupted, including the suspension of screening programmes. Our findings highlight the need to communicate changes in the availability of these services to FPs, as well as the need to develop and coordinate a plan for addressing the backlog in missed tests. In the P3 Model, practice-level factors describe setting-specific elements, such as the presence of structural barriers (e.g. reduced in-person visits during the COVID-19 pandemic). Our study identifies the need for decision support tools that prioritize preventative care visits for high-risk populations, electronic reminders to identify patients who require screening tests, and the need to create additional capacity to address the backlog in missed preventative care (e.g. evening clinics). These policy/practice changes have the potential to reduce structural barriers for preventative care during crisis situations, especially for groups experiencing marginalization who face added barriers to healthcare (beyond those they regularly encounter) during a pandemic.^29^ Provider-level factors relate to individual traits and capacities of the FP. Our study highlights FPs’ recognition of the importance of preventative care as well as the emotional impact of patient care issues (e.g. late diagnoses, worsening health status) that result from missed preventative care. Patient-level factors include patient-specific attributes that influence the utilization of preventative care. Study findings emphasize the need for disaster and pandemic response plans to provide patients with accurate information about available services and how patients can access tests once normal operations resume. Patients also need reassurance and education that accessing preventative care is important for their ongoing physical and mental health, and that visits to their FPs’ offices present minimal risk. As noted by the P3 Model, all factors that influence practice-, provider-, and patient-level components have the potential to subsequently influence patient outcomes.

The challenges associated with delivering preventative care during the COVID-19 pandemic also create a psychological burden on FPs, such as a moral injury—the “the psychological distress that results from actions, or the lack of them, which violate someone’s moral or ethical code.”^30^ FPs, as regulated professionals, are committed to providing ethically appropriate care. As per the Canadian Medical Association Code of Ethics and Professionalism, physicians are expected to take the required action to prevent or reduce the incidence of harm for their patients.^31^ Due to COVID-19, FPs may have felt that they involuntarily violated their ethics code when they were unable to order routine screening tests to inform plans of care and their professional commitment to prevention was altered. Moral injuries were compounded by increased workloads among FPs, resulting in growing burnout. National surveys completed in 2021 by Statistics Canada^32^ and the Canadian Medical Association^33^ reported that 46% and 59% of physicians, respectively, felt that their mental health status had worsened since the onset of the pandemic. As a result of burnout among FPs, many have reported plans to reduce their clinical presence/workload^33^ or leave/change jobs entirely (excluding retirement).^32^ These consequences have the potential to further reduce the availability of FPs, generating even more challenges when attempting to optimize preventative care in primary care. Therefore, while the cause of burnout and psychological decline among FPs is multifactorial, the inability to provide preventative care during the pandemic and the long-term implications for patients and providers has shown to be a contributing factor.

## Limitations and future research

Findings were collected across 4 regions in Canada, all of which differed in the policies and protocols that guided the delivery of preventative care by FPs during the pandemic, due to the provincial/territorial organization of healthcare systems. As a result, data may predominantly reflect select regions/provinces. As well, interviews with FPs were completed during different stages of the pandemic whereby COVID-19 incidence varied; the perceived burden of COVID-19 at the time of the interview may have influenced the reflections provided by FPs. In addition, team-based models of primary care allow for other providers (aside from FPs) (e.g. nurse practitioners) to deliver the bulk of preventative care. Future epidemiological studies are needed to examine the impacts of delayed/missed preventative care on short- and long-term health outcomes. As well, a more targeted exploration of this topic would be useful to gather a wider range and breadth of opinions and perspectives from FPs and other members of primary care teams (e.g. nurses, pharmacists).

## Conclusion

FPs in primary care faced many challenges when providing preventative care services during the COVID-19 pandemic. Preventative care has the potential to promote timely detection, treatment, and management of disease to optimize population health outcomes; therefore, we should aim to enhance future pandemic and disaster preparedness to ensure preventative care is sustained when health system disruptions occur. We identified practice-, provider-, and patient-level factors to include in planning for preventative care during health system crises, all of which call for coordinated actions between the laboratory and diagnostic services and primary care sectors.

## Data Availability

Data sharing is not available for the datasets generated or analysed during the current study for ethical and confidentiality reasons.
